# 3D chemical imaging of the brain using quantitative IR spectro-microscopy†
†Electronic supplementary information (ESI) available. See DOI: 10.1039/c7sc03306k


**DOI:** 10.1039/c7sc03306k

**Published:** 2017-10-17

**Authors:** Abiodun Ogunleke, Benoit Recur, Hugo Balacey, Hsiang-Hsin Chen, Maylis Delugin, Yeukuang Hwu, Sophie Javerzat, Cyril Petibois

**Affiliations:** a University of Bordeaux , Inserm U1029 LAMC , Allée Geoffroy Saint-Hilaire Bat. B2, F33600 Pessac , France . Email: cyril.petibois@u-bordeaux.fr ; Email: petibois@gate.sinica.edu.tw; b Academia Sinica , Institute of Physics , 128 Sec. 2, Academia Rd., Nankang , Taipei 11529 , Taiwan , Republic of China

## Abstract

Three-dimensional (3D) histology is the next frontier for modern anatomo-pathology. Characterizing abnormal parameters in a tissue is essential to understand the rationale of pathology development. However, there is no analytical technique, *in vivo* or histological, that is able to discover such abnormal features and provide a 3D distribution at microscopic resolution. Here, we introduce a unique high-throughput infrared (IR) microscopy method that combines automated image correction and subsequent spectral data analysis for 3D-IR image reconstruction. We performed spectral analysis of a complete organ for a small animal model, a mouse brain with an implanted glioma tumor. The 3D-IR image is reconstructed from 370 consecutive tissue sections and corrected using the X-ray tomogram of the organ for an accurate quantitative analysis of the chemical content. A 3D matrix of 89 × 10^6^ IR spectra is generated, allowing us to separate the tumor mass from healthy brain tissues based on various anatomical, chemical, and metabolic parameters. We demonstrate that quantitative metabolic parameters can be extracted from the IR spectra for the characterization of the brain *vs.* tumor metabolism (assessing the Warburg effect in tumors). Our method can be further exploited by searching for the whole spectral profile, discriminating tumor *vs.* healthy tissue in a non-supervised manner, which we call ‘spectromics’.

## Abstract 2

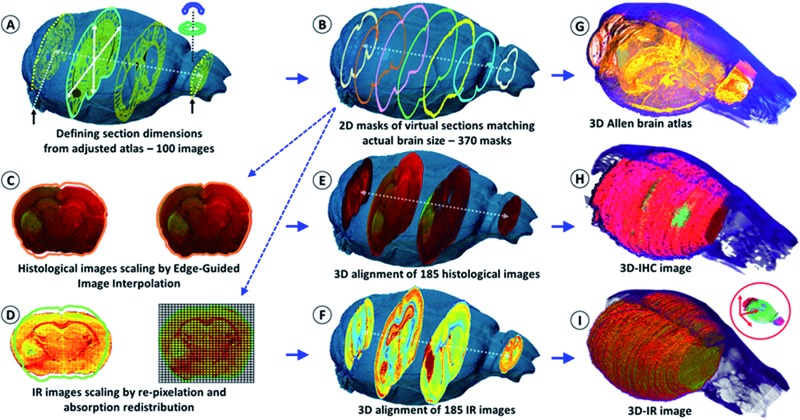
Here, we introduce a unique high-throughput infrared (IR) microscopy method that combines automated image correction and subsequent spectral data analysis for 3D-IR image reconstruction.

## Introduction

Three-dimensional (3D) microscopy is a powerful approach for imaging biological specimens.^1^ It offers excellent spatial resolution and facilitates the observation of tissue sub-structures and content^2^ under physiological and pathological conditions. However, although it is feasible to achieve a high spatial resolution close to the diffraction limit in small, transparent biological specimens^3^ such as cultured cells, it remains difficult to achieve high-resolution images of larger, more optically challenging entities such as tissue blocks, biopsies, or organs.^4^ 3D pathology is expected because tissue blocks are not naturally transparent, and they contain complex 3D networks (blood and lymph systems, membranes, nerves and other fibers, *etc.*), a 3D arrangement of different cell phenotypes that is not homogeneous, and an extracellular space that is composed of many other compounds and filamentous structures. From a geometric point of view, it is possible in principle to instantly visualize tissue abnormalities using 3D pathology and it has significant advantages compared to the usual 2D histology.^5^ However, labeling techniques are not quantitative approaches, and so 3D rendering cannot provide the distribution of the molecular concentrations.^4^ Access to the molecular concentrations will be crucial in overcoming the current interpretation limit of pathological features based mostly on anatomical rendering.

Several approaches have already been proposed for *ex vivo* 3D spectro-microscopy imaging, such as serial sections based electron,^6^ X-ray fluorescence,^7^ infrared,^2^,^8^ mass,^9^ and Raman^10^ microscopies, with a 3D reconstruction of small tissue volumes at the micro- and nano-scopic scales. Mass spectrometry (MS) imaging was the first spectroscopic technique that was able to create a 3D reconstruction of the chemical information of a tissue sample.^11^ In principle, mass spectra can provide thousands of signals recorded from each voxel of a 3D MS image. A wide variety of molecules can be imaged in this way, including proteins, peptides, lipids, and endogenous and exogenous metabolites, although they cannot be imaged all together. Thus, no global chemical information can be obtained from the sample (including proteins, lipids and sugars).

A common limit to all chemical imaging techniques that might be used for 3D volume rendering is that, at microscopic scale, 3D reconstruction is considerably more challenging relative to two-dimensional imaging because sectioning artifacts such as tissue tears, bends, folds, and cracks become unmanageable.^12^ Moreover, in 3D pathology, it is also mandatory to consider soft tissue distortions due to organ removal by surgery or biopsy. The shape of the tissue is considerably altered *via* cryomicrotomy, and the final 3D reconstruction model that is created from serial 2D sections will be significantly distant from reality. For quantitative analyses in 3D pathology, the determination of the molecular concentrations as well as the distribution of tissue sub-structures will be directly dependent on the recovery of the native 3D shape of the tissue/organ.^13^ As a general consideration, the lack of 3D imaging of the tissue/organ in its native shape is a major methodological issue for histological analyses, both in 2D and in 3D. To overcome that limitation in the development of 3D pathology methods, MRI,^14^ μ-CT,^13^ and ultrasound^15^*in vivo* imaging have been used to provide the actual volume of the tissue to analyze.

Here, we present an analytical methodology for developing a 3D quantitative pathology. Our experimental strategy is based on a combination of X-ray tomography for the *in vivo* acquisition of a 3D image of a mouse brain in the skull and infrared (IR) spectro-microscopy for the histological analysis. The results shown in this paper were obtained using a mouse brain in which glioma tumor cells had been implanted to grow a tumor in 28 days before the acquisition of 3D *in situ* and 2D histological images. The presence of a tumor in the brain was the perfect challenge for the quantitative chemical analysis of tissues, as tumors are highly different from healthy tissues in terms of chemical composition^16^ and metabolism.^17^

## Results and discussion

### Acquisition of the actual 3D shape of the brain

We first used the heads of mice for the X-ray tomographic analysis of the brain volume (see the video in the ESI, supplementary material 1†). The heads were analyzed with and without the brain inside the skull to obtain the actual brain volume *via* the subtraction of segmented 3D images. The segmentation method allowed us to obtain the meshing of the brain with a 2 μm accuracy (see the figures and videos in ESI, supplementary material 2 and 3†). The high-resolution images were used as models for resizing the low-dose 3-projection X-ray images. The objective was to obtain a 3D image of the brain without altering its content due to ionizing radiation, but at the same time, X-ray microscopy was also chosen to avoid the use of contrast agents or labeling methods (as used in MRI,^18^ PET/SPECT,^19^ intravital imaging,^20^*etc.*), which modify the chemical content of the sample. Therefore, it would have affected chemical analyses using IR microscopy after histology. The volume rendering from 3 axial absorption projections allowed us to obtain a realistic volume rendering of the mouse brain that was used to visualize all of the tissue sections and create a 2D mask of their planar limits. The main issue following the acquisition of a brain volume from 3 axial absorption projections was to determine the start and end points of brain sectioning as well as the actual axis of sectioning. This issue was solved using an available anatomical atlas of a mouse brain, the Allen Mouse Brain Atlas^21^,^22^ (see the ESI, supplementary material 4†). We resized the atlas with respect to the actual volume of the mouse brain. To this end, a graph-theoretic slice-to-slice reconstruction was performed with a global histology-to-CT reconstruction to achieve high accuracy, both in the alignment of the features between the slices and in the 3D shape of the reconstructed brain.

Methods have been proposed for the 3D reconstruction of tissue blocks, but the tissues were first stained^23^ or block-face photographic volume registration^5^ was used with MRI to help correct the shape in the soft tissue images. However, the use of labeling and staining methods or gadolinium injections for MRI prevent further “unaltered” chemical analysis. Our methodology overcomes such a bottleneck, providing both an image registration and correction method for reconstructing 3D tissue blocks, and can determine the molecular concentrations in 3D at microscopic resolution. The key advantage is the development of a genuine combination of *in vivo* 3D imaging with quantitative spectro-microscopy for producing a 3D quantitative chemical image of a tissue block. Our technique requires only a fresh-frozen tissue block to obtain a 3D chemical image.

### Acquisition of a 3D IR spectrum matrix of the brain

After the acquisition of the three X-ray projections for the volume rendering of the brain, the organ was removed from the skull and deposited in the upright position (with the cerebellum on the bottom, thus tamping the brain volume in the coronal axis) on the sample holder for continuous transverse cryomicrotomy at a 20 μm thickness. A series of 340–385 sections could be obtained depending on the organ size. Alternately, one section was stored for conventional histology (named histological images) and one section was processed for the IR microscopy analysis (named IR images). The goal was to obtain a series of histological sections that were sufficiently representative of the whole brain for comparative analyses using IR microscopy and histology, and for 3D image reconstruction. An example of a 2D IR image of a mouse brain with typical IR spectra from different anatomical regions is shown in the ESI, supplementary material 5.†

**Fig. 1:**
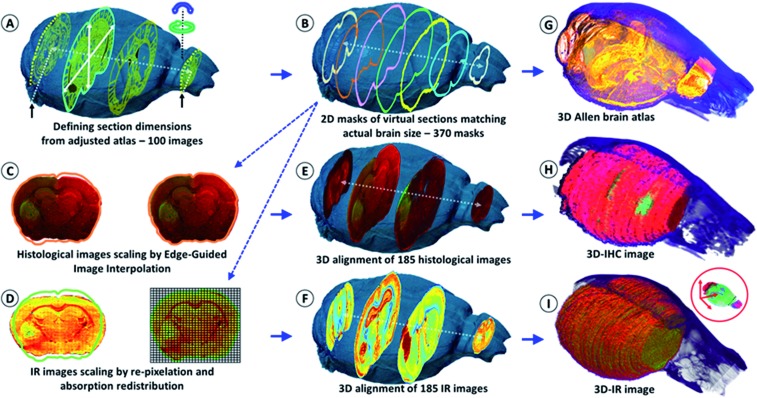
Schematic of the shape correction process for 2D images and 3D volume reconstructions. The actual volume model of the mouse brain is used to resize the anatomical images of the Allen Mouse Brain Atlas in (A). The first and last frames corresponded to the limits of the full set of tissue sections obtained using continuous cryomicrotomy (histological and IR – 370 sections). In (B), the virtual 2D masks of the 370 images are extracted from the actual volume of the mouse brain. (C) The histological images are corrected using edge-guided image interpolation. (D) The IR images are corrected *via* the re-pixelation and redistribution of the full spectral absorbance. The total spectral absorbance of the 2D IR image is calculated before and after IR spectra redistribution for the validation of the image correction process. (E) The 185 histological images are positioned in the actual volume model of the mouse brain for 3D alignment and patching. The alignment of each of the IHC images is performed using a global smoothed slice-shape morphing with a normalized redistribution of pixel values over the reached virtual slice surface (extracted from the X-ray model 2D-mask). (F) The 185 IR images are also aligned and patched according to the same procedure. (G–I) The 3D reconstructions of the Allen brain atlas, the 3D-IR image and the 3D-IHC image resized to match the actual dimensions of the mouse brain.

The IR spectra from different regions of the brain show very different absorption profiles, thus confirming that variations in the chemical content are significant. For the individual 2D-IR images, the (1800–900 cm^–1^) intensity scale ranged from 0–192 to 0–331 for the whole set of 185 images. The intensity scale was set as free for the 3D-IR image reconstruction. The first reconstruction of the 3D-IR image from 2D raw IR images (without any planar shape correction) was center-aligned using the central axis between the lobes as the anatomical reference. As shown in Fig. 1, the general shape of the 3D-IR image of the brain contained numerous distortions. The distortions came from organ shape alterations during surgery (due to the relapse of the brain volume once extracted from the skull, which exerts a pressure on brain tissues, and also due to the gravity-related collapse of this very soft tissue when deposited on the sample holder). This was also due to well-known tissue alterations during cryomicrotomy, where tears, bends, cracks, *etc.* appear at the tissue sectioning or deposition process. This clearly shows the relevance of using a 3D *in situ* (or *in vivo*) imaging method for obtaining a realistic volume rendering of a mouse brain before histological analyses. This is also critical for ensuring further quantitative chemical analysis from the 3D-IR image.

### Correction of the 2D histological images from the 3D X-ray tomogram

The IR and histological images were corrected for the shape alterations using the X-ray tomogram and 100 virtual slices (from 100 images – Fig. 1) given by the Allen Mouse Brain Atlas. We first compared the anatomical images with the IR and histological images to define the sectioning plan effectively used while obtaining all of the histological sections. The anatomical images are resized according to the actual 3D volume of the mouse brain. They serve as a global reference for comparing the anatomical regions observed from the visible, histological, and IR images. Importantly, they allow for the defining of the first and last sections obtained on a mouse brain using cryomicrotomy, of which the number varies according to the size of the organ and some potential loss of sections at the extremities. They are also used for proper alignment and 3D patching after shape corrections, as shown in Fig. 1.

The sets of IR and corresponding histological images (370 dual images) were used for brain volume rendering. The average deviation observed from the 2D image corrections was 11% in height and 8% in width. The 3D histological image was segmented to highlight the tumor (labeled, green fluorescence – see the ESI, supplementary material 6†). We can observe that the reconstructed 3D images of the brain perfectly matched the actual volume of the brain defined in the X-ray tomogram. We applied classical IR spectroscopy analysis to highlight the tumor from the 3D chemical image, *i.e.*, the protein-to-lipid ratio defined by the absorption ratio (1700–1480 cm^–1^)/(1760–1710 cm^–1^), or the absorption ratio between amide I and lipid esters.^24^–^26^ This analysis highlights the tumor mass, which contained a higher concentration of proteins and a lower concentration of lipids than its surrounding tissues.

### Anatomy of the brain based on 3D chemical data

**Fig. 2:**
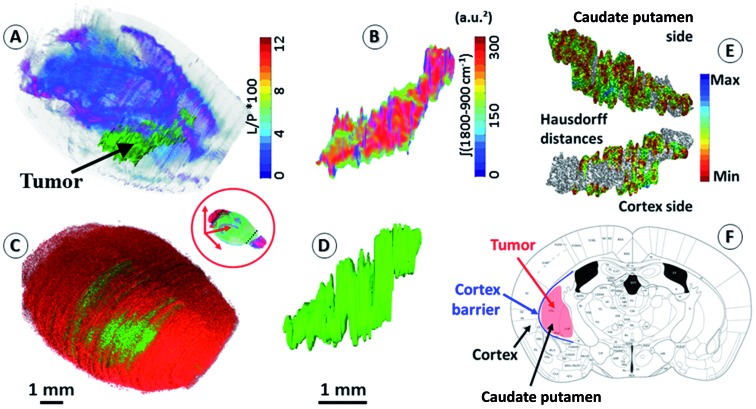
Segmentation of brain regions based on chemical content. (A) A 3D-IR image of the mouse brain in the skull highlighting the tumor mass using the [(1760–1710 cm^–1^)/(1700–1592 cm^–1^) × 100] absorption ratio (L/P as indicated in figure scale legend – videos are provided in the ESI, supplementary material 7 and 8†). With the tumor seen mostly in green, we can also distinguish the white matter distribution as the purple-blue part of the brain image. (B) A 3D-IR image of the tumor mass extracted from the whole brain *via* the segmentation of the voxels presenting the chemical profile of the tumor (full spectral absorbance). (C and D) Similar views of the tumor mass in the skull and extracted from the 3D-histological image (green channel segmentation for the tumor). (E) A representation of the Hausdorff distances between the 3D-histology (reference) image of the tumor mass and its 3D-IR counterpart (both sides of the tumor mass). (F) An illustration of the tumor growth mechanics as revealed by the segmentation of 3D-IHC and 3D-IR images of the tumor volume with respect to the invaded brain regions.

An important objective of our study was to demonstrate that 3D chemical imaging using IR spectro-microscopy can be used for the 3D pathological investigation of large tissue blocks. We segmented the tumor mass based on a simple spectral analysis to compare it with the tumor volume rendering from the 3D histological image (see the ESI, supplementary material 7 and 8†). As expected, the IR spectra extracted from the tumor and at a similar location in the left hemisphere show important differences for most of the absorption regions. The shape of the tumor volume was found to be very similar between the IR and histological analyses, with the calculation of the Hausdorff distances^27^ between the two volumes showing only marginal differences in the global shape of the tumor, but a significant difference in volume (3D-IR = 9.39 mm^3^; 3D-IHC = 12.24 mm^3^; difference ∼30%). The ability to extract the 3D spectrum matrix of the tumor mass is important to specifically analyze its chemical content. This is also true for healthy brain tissues (at least the left hemisphere, which is not affected by the tumor metabolism and the mechanical pressure it exerts on the surrounding tissues). Fig. 2 shows 3D-IHC and 3D-IR image examples of the segmentation performed on the mouse brain, both for the tumor mass extraction and for defining anatomical structures from chemical analyses on the 3D-IR image. This segmentation can be based on the absorption profiles extracted from the spectra (as for the tumor mass with the lipid/protein contrast). It is worth noting that healthy brain tissues and anatomical entities can be successfully separated directly from the IR spectra. Therefore, an anatomical atlas of the mouse brain can be developed from its 3D quantitative chemical image.

The distinction between white and gray matter is similar to previous studies.^28^ The Hausdorff distance calculation reveals further interesting features (see the ESI, supplementary material 8†). The 3D-IHC image of the tumor shows the tissue volume occupied by the tumor cells. Conversely, the 3D-IR image shows the tissue volume, of which the chemistry is significantly altered by the tumor cells. The 30% difference between the two volumes represents the tissue volume where the tumor cells are present in the 3D-IHC image but where they did not significantly alter the chemical composition of the tissue, as revealed by the 3D-IR image.^24^ Thus, the difference is related to tissue areas where the tumor cells are dispersed. On the tumor side in front of the cortex, the differences between the IHC and IR images are very limited because the tumor is massive. Therefore, the tumor was blocked by the cortex barrier (except along the “tunnel” formed by the needle when implanting the tumor cells). On the opposite side, the differences between the IHC and IR images are more significant because the tumor is more diffuse. This is showing that the tumor had an easier way to invade the parenchyma through the caudate putamen region. This result illustrates the importance of 3D histology for understanding the anatomical–mechanical–chemical features that drive the growth of a tumor.

### Quantitative 3D metabolic images based on 3D chemical data

**Fig. 3:**
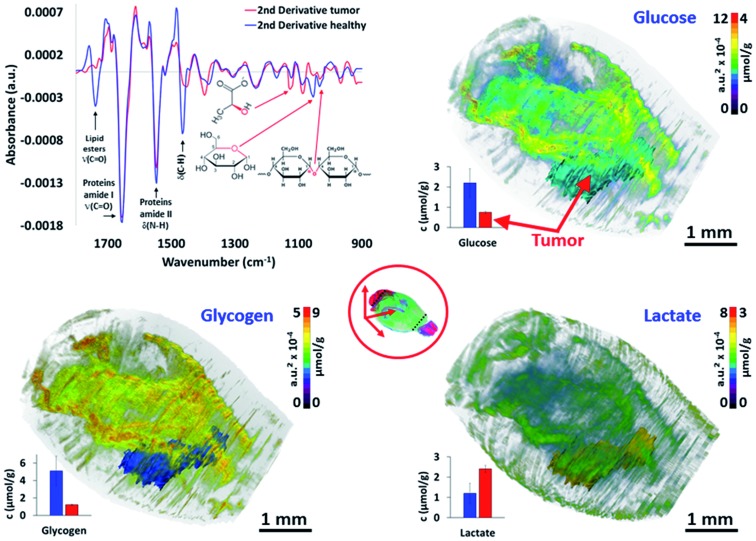
Quantitative metabolic analysis of the brain. From the 2^nd^ derivative of the 3D spectrum matrix, the glucose, glycogen and lactate IR absorptions are quantified and mapped for the whole brain. The 3D volume renderings with molecular concentrations have been determined according to the healthy brain (left hemisphere) as a reference (3D renderings are shown in the ESI, supplementary material 9–11†). The images are scaled with equivalent absorption (a.u.^2^ × 10^–4^) and concentration (μmol g^–1^) values.

The last major objective of this study was to demonstrate that 3D IR spectro-microscopy can achieve a quantitative molecular analysis of tissues. With the example of a tumor, the challenge was to analyze the major metabolic parameters of the brain:^29^ the glycogen, glucose and lactate concentrations. To obtain a quantitative analysis, we performed absorption integration from the most specific IR bands of glucose^26^,^30^ (1031 cm^–1^), glycogen^31^,^32^ (1024 and 1152 cm^–1^), and lactate^31^,^33^ (1127 cm^–1^) on all of the IR spectra of the 3D-IR image of the brain after calculating their 2^nd^ derivative, which is a standard procedure for fast IR spectral data extraction.^34^ From 2^nd^ derivative spectra, the 3D mapping of the molecular concentrations in the brain was found to be consistent between the tissue sections and thus no major contrast aberration came to alter the visual rendering of these analyses (Fig. 3 and the ESI, supplementary material 9–11†).

We first compared the molecular concentrations between enzymatic assays and IR spectra data analyses. A group of 10 mice with a similar tumor mass in the right hemisphere were sacrificed to analyze their left hemisphere. Fluorescence enzymatic assays^35^ made on tissue homogenates provided 2.2 ± 0.7 μmol g^–1^ of glucose, 5.1 ± 1.7 μmol g^–1^ of glycogen, and 1.2 ± 0.5 μmol g^–1^ of lactate. These results are consistent with other studies on mice^36^–^39^ that have been reported from similar enzymatic assays. Interestingly, the regional distribution of glucose^36^ in mice brains have been found to vary, with a 3-fold amplitude; similarly, a 2-fold variation amplitude was found for lactate^40^ and glycogen.^38^,^41^ Such concentration variations per brain region were also found in our 3D-IR images. When we considered the distribution of absorption intensities for glucose, glycogen, and lactate IR absorption, we observed that 95% of spectra ranged within a 2-3-fold intensity scale value. Extreme values were rejected from the calculations (notably the 0 values, representing 86% of rejected spectra, which were probably due to noisy signal or a distorted baseline preventing the use of the 2^nd^ derivative spectra absorption). The spectra included in this study (>95% for each anatomical region) allowed us to define a distribution of concentrations in accordance with the enzymatic assays performed on the brain regions, with the glucose scale ranging from 1.3 to 3.5 μmol g^–1^, glycogen from 3.6 to 8.4 μmol g^–1^, and lactate from 0.7 to 1.9 μmol g^–1^ (in other words, each metabolite has a 2-3-fold concentration variation in the brain).

These concentration variations in the mouse brain are consistent with previous studies,^36^,^39^,^41^ although they are not fully comparable since the microscopic studies of the metabolic concentrations in the brain have never been done previously on fresh – snap-frozen only – tissues. It is also important to note that the tumor exhibited a significantly lower glucose (0.8 ± 0.1 μmol g^–1^; *P* < 0.05) and glycogen (1.2 ± 0.1 μmol g^–1^; *P* < 0.05) concentrations compared to their healthy tissue counterpart in the left hemisphere (considering similar locations – see the ESI, supplementary material 12†), while the lactate concentration was much higher (2.4 ± 0.2 μmol g^–1^; *P* < 0.05). With respect to the healthy brain tissues, these metabolic changes in the tumor mass are typical of a Warburg effect, where glycolysis is increased and thus depletes the glucose–glycogen stores and consequently raises the production of lactate as a by-product.^42^ However, the distributions of the metabolic concentrations in the tumor mass were found to be very homogeneous, which is also characteristic for that size of glioma solid tumor.^43^ Importantly, we tried other resolutions (5, 10, and 50 μm), and it appeared that 5 μm thick sections did not provide a reliable S/N for the quantitative analyses.^44^ The 10 μm resolution was not reliable for 3D image reconstruction as too many sections were lost from the continuous sectioning, and the 50 μm resolution was insufficient to provide all of the details found at 20 μm resolution. Thus, the 20 μm resolution appears as the best compromise for large 3D-IR image reconstruction with the current IR microscopy technology.

Until now, quantitative histology remained limited to the quantitation of objects^45^ and sub-structures^46^ in tissue sections, but not to its chemical content.^47^ Here, we combined an efficient anatomical rendering of the mouse brain with a quantitative chemical analysis of its contents from the same dataset. The 3D volume renderings for anatomical, chemical and metabolic content of the mouse brain with a tumor reached an incomparable level of information for 3D microscopy analysis. As we demonstrated for the anatomical and metabolic features of a mouse brain, the exploitation of the whole spectral information, called ‘spectromics’, from a tissue block will tremendously expand the possible applications of this methodology.

## Conclusions

We used a combination of 3D *in vivo* and histological imaging techniques to achieve a quantitative 3D chemical analysis of the mouse brain. The most amazing feature of this methodology is that it can resolve and co-register anatomical and metabolic parameters from the same dataset, without the use of any reagent or imaging contrast agent. Furthermore, the IR spectroscopy technique used is far less complex than other spectroscopies, and direct exploitation of spectral data for producing relevant 3D anatomical, chemical and metabolic images can be achieved. Our approach includes high-fidelity 3D imaging and automated mathematical analysis performed on a whole organ, promising deep impact on biomedical knowledge and research.

## Materials and methods

### Sample preparation

The 3D image model of the mouse brain used for the 2D and 3D-IR image corrections was obtained using healthy animals (10 to 12 week-old male rag-γ2C^–/–^ immunodeficient mice). The study on the animals was approved by the local ethics committee (University of Bordeaux) under the agreement no. 501305-1, and all of the experiments were performed in compliance with the relevant laws and institutional guidelines for animal welfare. A series of 12 animals were used for the acquisition of the 3D image of the head after dissection. The dissection consisted in the removal of the skin, eyes, tongue, teeth, *etc.* to obtain the skull and the brain sample. The X-ray tomographic images were obtained in sequence with and without the brain (360 projections over 180 degrees, providing a 2 μm resolution meshing of the brain). For the skull imaging, the brain was aspirated through the occipital hole. The internal part of the skull was further cleaned from possible tissue remains *via* enzymatic digestion (Liberase TL, Roche ref. 05401020001) for 20 minutes at 37 °C. The low-dose X-ray images of the brain further used for the IR analyses were obtained using 3 projections (coronal, sagittal and axial views) under the same conditions as for the 360 projection images, thus limiting the X-ray dose to a negligible amount (not heating the brain before the histological analyses). The aim was to reconstruct a 3D model of the brain with limited 2D projections using the high-resolution X-ray tomographic images as references.

The mouse brains that were prepared for the 3D histological analyses were xenografted with NCH421K glioma tumor spheroids (proneural, stem-like cells). In brief, primary tumor-derived NCH421K spheroids (5 spheroids of 10^4^ cells per mouse) were implanted into the right cerebral cortex using a Hamilton syringe fitted with a needle (Hamilton, Bonaduz, Switzerland) following a procedure that was previously described.^48^ Injections were realized in the striatum (2.2 mm on left from bregma 0 and 3 mm depth) using Hamilton syringe. Full brains (with a xenografted tumor on one lobe and healthy brain on the other lobe) were removed from the sacrificed mice after 28 days of tumor growth. The sample holder with brain were inserted into a plastic tube and plunged into liquid N_2_ for instant freezing. The frozen brain was deposited in the upright position (with the cerebellum on the bottom) on cooled glue (polyvinyl alcohol for cryostat, –20 °C) to avoid tissue embedding. The total duration from the death of animal to the complete freezing of brain was always less than two minutes, and guaranteed that degradation in brain cell and tissue contents was limited. A complete sectioning of the brain was performed at 20 μm thickness (Cryostat CM1900, Leica-Microsystems, France). A total of 340–385 sections was obtained depending on the brain dimensions (and where the sectioning was stopped in the cerebellum mass). The tissue slides were maintained at –80 °C before IR and IHC image acquisition. For histological imaging, all of the tissue sections were incubated with antibodies against human vimentin antigens (Santa Cruz 6260) and a green fluorescent secondary antibody (goat anti-mouse 488 antibody, Interchim FP-SA4010). Imaging was carried out using a Nikon eclipse E600 microscope.

### X-ray image acquisition

Microradiology was performed with unmonochromatized (white) synchrotron X-rays emitted at the 01-A beamline wavelength shifter of the National Synchrotron Radiation Research Center (NSRRC, Hsinchu, Taiwan). The photon energy ranged from 4 keV to 30 keV with a peak intensity of ∼12 keV; the beam current was kept constant at 360 mA with the top-up operation mode over all acquisition periods. To obtain 4.59 × 3.43 mm images, X-rays were converted into visible light using a CdWO_4_ single crystal scintillator and then the photons were detected using an optical microscope equipped with a 1600 × 1200 pixel CCD camera (model 211, Diagnostic Instruments). We reduced the radiation dose by attenuating the X-ray beam with two 550 μm silicon wafers. The dose was 33.9 Gy per 100 ms for a specimen thickness of 1 cm placed before the sample. The sample-scintillator distance was 5 cm. We used a 2× lens in the optical microscope to obtain the desired field of view; the pixel size in the final image was 2 × 2 μm^2^. A simple background flattening image filter was used for the large area micro-radiology images. The conceptual details of synchrotron-based microtomography, including the absorption and phase contrast, have been discussed in a previous study.^49^ The high-resolution tomographic images were captured at 360 angles over 180 degrees. The low resolution (and low X-ray dose) images were captured with 3 angles (coronal, sagittal and axial views) and reconstructed using the high-resolution models (see the ESI, supplementary material 1–3†).

### IR acquisition for 20 μm spatial resolution imaging

We analyzed the mouse brain tissue sections using IR microscopy. The QCL-IR microscope (Spero®, DRS Daylight Solutions, CA, USA) is equipped with 4 IR lasers providing wavelengths every 4 cm^–1^ along the 1800–900 cm^–1^ spectral interval, thus 225 absorption values. The microscope is constantly purged with dry air and the sample compartment is isolated from ambient air using a plastic box. The detector is a non-N_2_-liquid frozen focal plane array (FPA) detector with 480 × 480 elements. The 20 μm pixel size was obtained after acquisition by binning 5 × 5 pixels. The IR image acquisitions lasted up to 2 hours per section at the largest tissue section dimensions, ∼6 × 8 mm. A total of 170–190 IR images was obtained per brain for the 3D-IR image reconstruction (the same number as for the corresponding histological image, providing a comparison between tumor volumes). The microscope was installed in a thermally controlled room (20 °C) for standardizing the ambient conditions during acquisition over the total duration of the acquisitions (2 months).

### 3D IR and 3D histological images reconstruction

The 2D- and 3D-IR images presented in this article come from a mouse brain with 370 sections (see the ESI, supplementary material 6 and 7†). The 2D-IR images were obtained and represented a matrix of 9.4-million IR spectra and 250 GB of raw data on a storage server (reduced to 10 GB after processing with 5 × 5 pixel binning). The same number of histological sections was further obtained. The visible images of the histological sections were coupled to the 2D masks of the virtual brain sections extracted from the 3D X-ray tomogram of the mouse brain for further correction and resizing (Fig. 1). The resizing of the 2D-histological images was done using edge-guided image interpolation. The first and last sections obtained *via* cryomicrotomy are marked with black arrows in Fig. 1A. The 2D-mask of each image was extracted from the actual volume of the mouse brain (representing a 20 μm distance between the 2D-masks to match the thickness of the histological sections). The lacking 2D-masks (370 sections *vs.* 100 images in the Allen brain atlas) were completed for the correction of all of the tissue sections. The histological images were corrected for shape alteration using edge-guided image interpolation with their corresponding 2D-mask for reference (Fig. 1). The IR images were corrected *via* the re-pixelation and redistribution of the full spectral absorbance at the 2D image level. A pixel grid at a resolution of the IR image (which here was 20 μm lateral resolution) covers the 2D-mask that the 2D-IR image must match. The IR spectra contained in the IR image pixels are redistributed in the pixel grid of the 2D-mask. The total spectral absorbance of the 2D-IR image is calculated before and after the IR spectra redistribution to ensure that the chemical information of the tissue sections remained unchanged. The histological images were positioned in the actual volume model of the mouse brain for 3D alignment and patching. The alignment was performed for the recognition of various anatomical patterns with respect to the Allen brain atlas images in Fig. 1A and the 2D-masks obtained in Fig. 1B. Typical anatomical features are salient angles found at the surface of brain volume, such as the longitudinal cerebral fissure, the lobe-cerebellum interfaces, *etc.* The 2D-IR images were also aligned and patched according to the same procedure as for histological images.

### Brain metabolic assays

In a series of 10 mice with 18–23 days of tumor development in the right hemisphere, the brains were harvested for the dissection of the left hemisphere (healthy tissue). The tissues were immediately weighed and brain homogenates were obtained *via* sonication in 10% wt/vol of 0.1 M NaOH and 0.01% SDS and centrifuged for 15 min at 16 000*g* at 4 °C. The supernatant was acidified and diluted with 0.03 M HCl. Glycogen and glucose were measured with a fluorescence enzymatic assay using the amyloglucosidase method.^35^ Glycogen was digested with amylo-α-1,4-α-1,6-glucosidase (AG) (Sigma). The glucose levels were determined with hexokinase and glucose-6-phosphate dehydrogenase (Sigma) through the formation of NADPH from the reduction of NADP^+^. The glucose levels obtained from the samples without AG were subtracted from the samples with AG to determine the glycogen levels. The glycogen and glucose were both expressed as micromoles per gram of fresh brain tissue (μmol g^–1^). The effects of SD were evaluated using *t*-tests. For lactate, brain homogenates (20 mg) were added to 100 μl of ice-cold 3 M perchloric acid, homogenized using a homogenizer, and then centrifuged at 1000*g* for 5 min at 4 °C. The resulting supernatant was mixed with buffer containing glycine, hydrazine, and NAD, and was then added to LDH. The fluorescence measurements were taken at 350 nm excitation and 450 nm emission. The lactate concentration was calculated from a standard curve. For comparison with the IR spectral data, statistical tests were considered significant if *P* < 0.05.

### IR spectra data treatment

#### 3D-IR image reconstruction

The (1800–900 cm^–1^) spectral intensity integration was calculated for all of the IR spectra and a 3D image was reconstructed (the full spectral intensity 3D image of the brain). The full spectral intensity typically ranges between 0 and 300, and this scale was applied to all of the 2D-IR images before 3D reconstruction. The 3D patch of 2D-IR images was first performed with uncorrected 2D-IR images (see the ESI, supplementary material 13†) to show the mediocre volume rendering induced by the multiple tissue section shape alterations due to surgery, sample deposition on the sample holder, and cryomicrotomy. The same 3D patch performed with corrected 2D-IR images (see the ESI, supplementary material 14†) using X-ray imaging allowed us to properly align each 2D-IR image according to observable external anatomical details as explained above. Therefore, although the spectra data treatment was applied to the 2D-IR images, the positioning of each 2D-IR image in the 3D alignment was fixed for all of the volume renderings.

#### Tumor volume

We performed the segmentation and meshing of the tumor volume after classical spectroscopic analysis, *i.e.*, by calculating the protein-to-lipid absorption ratio (1700–1480 cm^–1^)/(1760–1710 cm^–1^). The mesh of the tumor volume is extracted as an independent volumetric image for shape comparison with its 3D-histological image (Fig. 2 and ESI, supplementary material 8†). The meshed tumor volumes, both the IR and histological, were subtracted as poly-surfaces to check the relevance of using the protein-to-lipid absorption ratio to reveal a glioma tumor in the brain. The difference between the IR and histological meshed volumes was measured and expressed as a percentage of the histological (reference) volume. The difference between meshed volumes of the tumor from uncorrected and corrected 3D-IR and 3D-histological images was also calculated to show the effect of 2D image correction on tumor volume rendering.

#### Quantitative metabolic analyses

Several metabolic parameters (glucose, glycogen, and lactate) were quantified in the 2D-IR images and the distribution of the concentrations (shown in Fig. 3 & ESI, supplementary material 9–12†) was determined as follows:

1- Second derivative IR spectra were calculated for the whole 3D spectrum matrix of the mouse brain. The IR spectra of the left hemisphere (healthy tissues) and for the tumor (from its meshed volume) were analyzed separately.

2- The absorption of glucose^26^,^30^ (1031 cm^–1^), glycogen^31^,^32^ (1024 and 1162 cm^–1^), and lactate^31^,^33^ (1127 cm^–1^) were measured by integrating band areas on the second derivative spectra (glucose: 1040–1027 cm^–1^; glycogen: 1027–1018 and 1167–1157 cm^–1^; and lactate: 1135–1114 cm^–1^ – expressed in a.u.^2^ × 10^–4^);

3- Since there is no histological method for determining the concentration of these metabolic molecules on histological sections, calibration of the molecular concentrations was established by considering the average value of their absorption as equivalent to the values of the metabolic assays on the brain homogenates obtained in parallel with another batch of 10 mice (same sex, age and experimental conditions, with a glioma tumor implanted the same day as for mice used in the histological/IR experiments). The calibration was performed using only the left hemisphere part of the 2D-IR images (the healthy tissues not affected by the tumor). The scaling of the molecular concentrations was done according to the distribution of the IR absorption for each molecular absorption (a.u.^2^ × 10^–4^ ± SD *vs.* μmol g^–1^ → μmol g^–1^ ± SD). The normal distribution of absorption (mean ± 3 × SD, in a.u.^2^ × 10^–4^ – data not shown) was calculated for the left hemisphere of the brain and for the tumor volume to reveal the heterogeneity of the metabolic parameters between the anatomical regions (mean ± 3 × SD, in μmol g^–1^).

4- The IR spectra corresponding to the anatomical regions meshed from the left hemisphere were analyzed separately (averaged spectra are shown in the ESI, supplementary material 12†) to reveal the mean ± SD values of the metabolic parameters and these were plotted (12 examples provided).

## Competing financial interests

The authors declare no competing financial interests.

## Supplementary Material

Supplementary movie

Supplementary information

Supplementary movie

Supplementary movie

Supplementary information

Supplementary information

## References

[cit1] Fratini M., Bukreeva I., Campi G., Brun F., Tromba G., Modregger P., Bucci D., Battaglia G., Spano R., Mastrogiacomo M., Requardt H., Giove F., Bravin A., Cedola A. (2015). Sci. Rep..

[cit2] Bobroff V., Chen H. H., Delugin M., Pineau R., Javerzat S., Petibois C. (2017). J. Biophotonics.

[cit3] Fernandez-Suarez M., Ting A. Y. (2008). Nat. Rev. Mol. Cell Biol..

[cit4] Stille M., Smith E. J., Crum W. R., Modo M. (2013). J. Neurosci. Methods.

[cit5] Vandenberghe M. E., Herard A. S., Souedet N., Sadouni E., Santin M. D., Briet D., Carre D., Schulz J., Hantraye P., Chabrier P. E., Rooney T., Debeir T., Blanchard V., Pradier L., Dhenain M., Delzescaux T. (2016). Sci. Rep..

[cit6] Mikula S., Denk W. (2015). Nat. Methods.

[cit7] Bourassa D., Gleber S. C., Vogt S., Yi H., Will F., Richter H., Shin C. H., Fahrni C. J. (2014). Metallomics.

[cit8] Wood B. R., Bambery K. R., Evans C. J., Quinn M. A., McNaughton D. (2006). BMC Med. Imaging.

[cit9] Kompauer M., Heiles S., Spengler B. (2017). Nat. Methods.

[cit10] Yao S., Chen H. H., Harte E., Ventura G. D., Petibois C. (2013). Anal. Bioanal. Chem..

[cit11] Ye H., Greer T., Li L. (2011). Bioanalysis.

[cit12] Clarke G. M., Murray M., Holloway C. M., Liu K., Zubovits J. T., Yaffe M. J. (2012). Int. J. Breast Cancer.

[cit13] Gregor T., Kochová P., Eberlová L., Nedorost L., Prosecká E., Liška V., Mírka H., Kachlík D., Pirner I., Zimmermann P., Králíčková A., Králíčková M. and Tonar Z., in Injury and Skeletal Biomechanics, Intech, 2012.

[cit14] Jones A. R., Overly C. C., Sunkin S. M. (2009). Nat. Neurosci..

[cit15] Schalk S. G., Postema A., Saidov T. A., Demi L., Smeenge M., de la Rosette J. J., Wijkstra H., Mischi M. (2016). Comput. Med. Imag. Graph..

[cit16] Mourant J. R., Yamada Y. R., Carpenter S., Dominique L. R., Freyer J. P. (2003). Biophys. J..

[cit17] Keckesova Z., Donaher J. L., De Cock J., Freinkman E., Lingrell S., Bachovchin D. A., Bierie B., Tischler V., Noske A., Okondo M. C., Reinhardt F., Thiru P., Golub T. R., Vance J. E., Weinberg R. A. (2017). Nature.

[cit18] Engelhorn T., Eyupoglu I. Y., Schwarz M. A., Karolczak M., Bruenner H., Struffert T., Kalender W., Doerfler A. (2009). Neurosci. Lett..

[cit19] Toyama H., Ichise M., Liow J. S., Modell K. J., Vines D. C., Esaki T., Cook M., Seidel J., Sokoloff L., Green M. V., Innis R. B. (2004). Int. Congr. Ser..

[cit20] Gleave J. A., Lerch J. P., Henkelman R. M., Nieman B. J. (2013). PLoS One.

[cit21] Lein E. S., Hawrylycz M. J., Ao N., Ayres M., Bensinger A., Bernard A., Boe A. F., Boguski M. S., Brockway K. S., Byrnes E. J., Chen L., Chen L., Chen T. M., Chin M. C., Chong J., Crook B. E., Czaplinska A., Dang C. N., Datta S., Dee N. R., Desaki A. L., Desta T., Diep E., Dolbeare T. A., Donelan M. J., Dong H. W., Dougherty J. G., Duncan B. J., Ebbert A. J., Eichele G., Estin L. K., Faber C., Facer B. A., Fields R., Fischer S. R., Fliss T. P., Frensley C., Gates S. N., Glattfelder K. J., Halverson K. R., Hart M. R., Hohmann J. G., Howell M. P., Jeung D. P., Johnson R. A., Karr P. T., Kawal R., Kidney J. M., Knapik R. H., Kuan C. L., Lake J. H., Laramee A. R., Larsen K. D., Lau C., Lemon T. A., Liang A. J., Liu Y., Luong L. T., Michaels J., Morgan J. J., Morgan R. J., Mortrud M. T., Mosqueda N. F., Ng L. L., Ng R., Orta G. J., Overly C. C., Pak T. H., Parry S. E., Pathak S. D., Pearson O. C., Puchalski R. B., Riley Z. L., Rockett H. R., Rowland S. A., Royall J. J., Ruiz M. J., Sarno N. R., Schaffnit K., Shapovalova N. V., Sivisay T., Slaughterbeck C. R., Smith S. C., Smith K. A., Smith B. I., Sodt A. J., Stewart N. N., Stumpf K. R., Sunkin S. M., Sutram M., Tam A., Teemer C. D., Thaller C., Thompson C. L., Varnam L. R., Visel A., Whitlock R. M., Wohnoutka P. E., Wolkey C. K., Wong V. Y. (2007). Nature.

[cit22] Yushkevich P. A., Avants B. B., Ng L., Hawrylycz M., Burstein P. D., Zhang H. and Gee J. C., in Biomedical Image Registration. WBIR 2006, ed. J. P. W. Pluim, B. Likar and F. A. Gerritsen, Springer, Berlin, 2004, vol. 4057.

[cit23] Roberts N., Magee D., Song Y., Brabazon K., Shires M., Crellin D., Orsi N. M., Quirke R., Quirke P., Treanor D. (2012). Am. J. Pathol..

[cit24] Noreen R., Moenner M., Hwu Y., Petibois C. (2012). Biotechnol. Adv..

[cit25] Petibois C., Desbat B. (2010). Trends Biotechnol..

[cit26] Petibois C., Déléris G. (2006). Trends Biotechnol..

[cit27] Fyllingen E. H., Stensjoen A. L., Berntsen E. M., Solheim O., Reinertsen I. (2016). PLoS One.

[cit28] Bambery K. R., Schultke E., Wood B. R., Rigley MacDonald S. T., Ataelmannan K., Griebel R. W., Juurlink B. H., McNaughton D. (2006). Biochim. Biophys. Acta.

[cit29] Vlashi E., Lagadec C., Vergnes L., Matsutani T., Masui K., Poulou M., Popescu R., Della Donna L., Evers P., Dekmezian C., Reue K., Christofk H., Mischel P. S., Pajonk F. (2011). Proc. Natl. Acad. Sci. U. S. A..

[cit30] Petibois C., Rigalleau V., Melin A. M., Perromat A., Cazorla G., Gin H., Deleris G. (1999). Clin. Chem..

[cit31] Hackett M. J., Sylvain N. J., Hou H., Caine S., Alaverdashvili M., Pushie M. J., Kelly M. E. (2016). Anal. Chem..

[cit32] Banerjee S., Pal M., Chakrabarty J., Petibois C., Paul R. R., Giri A., Chatterjee J. (2015). Anal. Bioanal. Chem..

[cit33] Petibois C., Cazorla G., Cassaigne A., Deleris G. (2001). Clin. Chem..

[cit34] Goormaghtigh E., Gasper R., Benard A., Goldsztein A., Raussens V. (2009). Biochim. Biophys. Acta.

[cit35] Gip P., Hagiwara G., Ruby N. F., Heller H. C. (2002). Am. J. Physiol.: Regul., Integr. Comp. Physiol..

[cit36] Shimada M., Kihara T., Watanabe M., Kurimoto K. (1977). Neurochem. Res..

[cit37] Swanson R. A., Choi D. W. (1993). J. Cereb. Blood Flow Metab..

[cit38] Sagar S. M., Sharp F. R., Swanson R. A. (1987). Brain Res..

[cit39] Horn T., Klein J. (2010). Neurochem. Int..

[cit40] Takimoto M., Hamada T. (1985). J. Appl. Physiol..

[cit41] Franken P., Gip P., Hagiwara G., Ruby N. F., Heller H. C. (2003). Am. J. Physiol.: Regul., Integr. Comp. Physiol..

[cit42] Liberti M. V., Locasale J. W. (2016). Trends Biochem. Sci..

[cit43] Vartanian A., Singh S. K., Agnihotri S., Jalali S., Burrell K., Aldape K. D., Zadeh G. (2014). Neuro-Oncology.

[cit44] Marcelli A., Cricenti A., Kwiatek W. M., Petibois C. (2013). Biotechnol. Adv..

[cit45] Chen H. H., Chien C. C., Petibois C., Wang C. L., Chu Y. S., Lai S. F., Hua T. E., Chen Y. Y., Cai X., Kempson I. M., Hwu Y., Margaritondo G. (2011). J. Nanobiotechnol..

[cit46] Zudaire E., Gambardella L., Kurcz C., Vermeren S. (2011). PLoS One.

[cit47] Petibois C. (2010). Anal. Bioanal. Chem..

[cit48] Drogat B., Bouchecareilh M., Petibois C., Déléris G., Chevet E., Bikfalvi A., Moenner M. (2007). J. Cell. Physiol..

[cit49] Meuli R., Hwu Y., Je J. H., Margaritondo G. (2004). Eur. Radiol..

[cit50] Campos B. (2010). Clin. Cancer Res..

