# Supplementary figures and images for: 3D chemical imaging of the brain using quantitative IR spectro-microscopy
Source: Chem Sci. 2017 Oct 17;9(1):189–98. doi: 10.1039/c7sc03306k (PMC5869290; doi:10.1039/c7sc03306k)

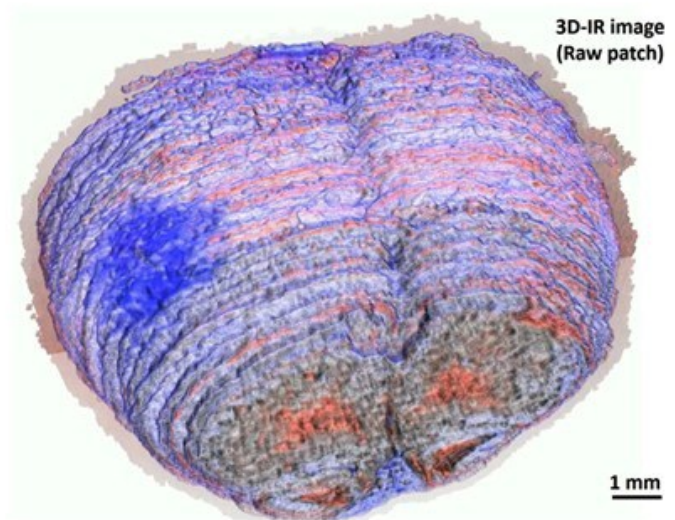

**Supplementary material 13**

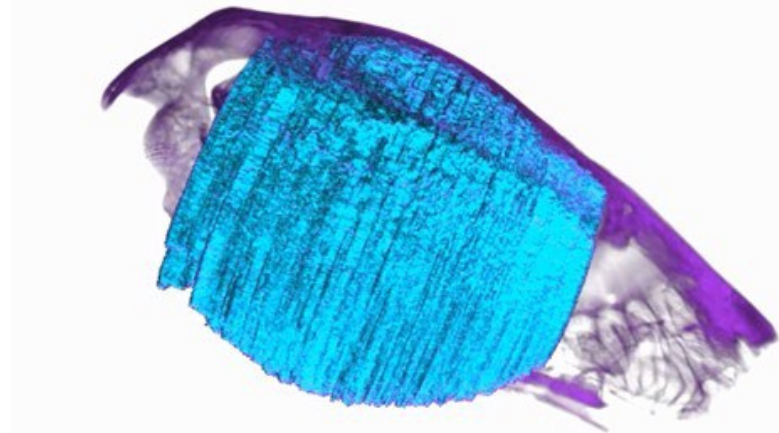

**Supplementary material 14**

**3D-IR image  
(corrected)**

**1 mm**

Supplement: Supplementary file 2 [file SC-009-C7SC03306K-s002.pdf]

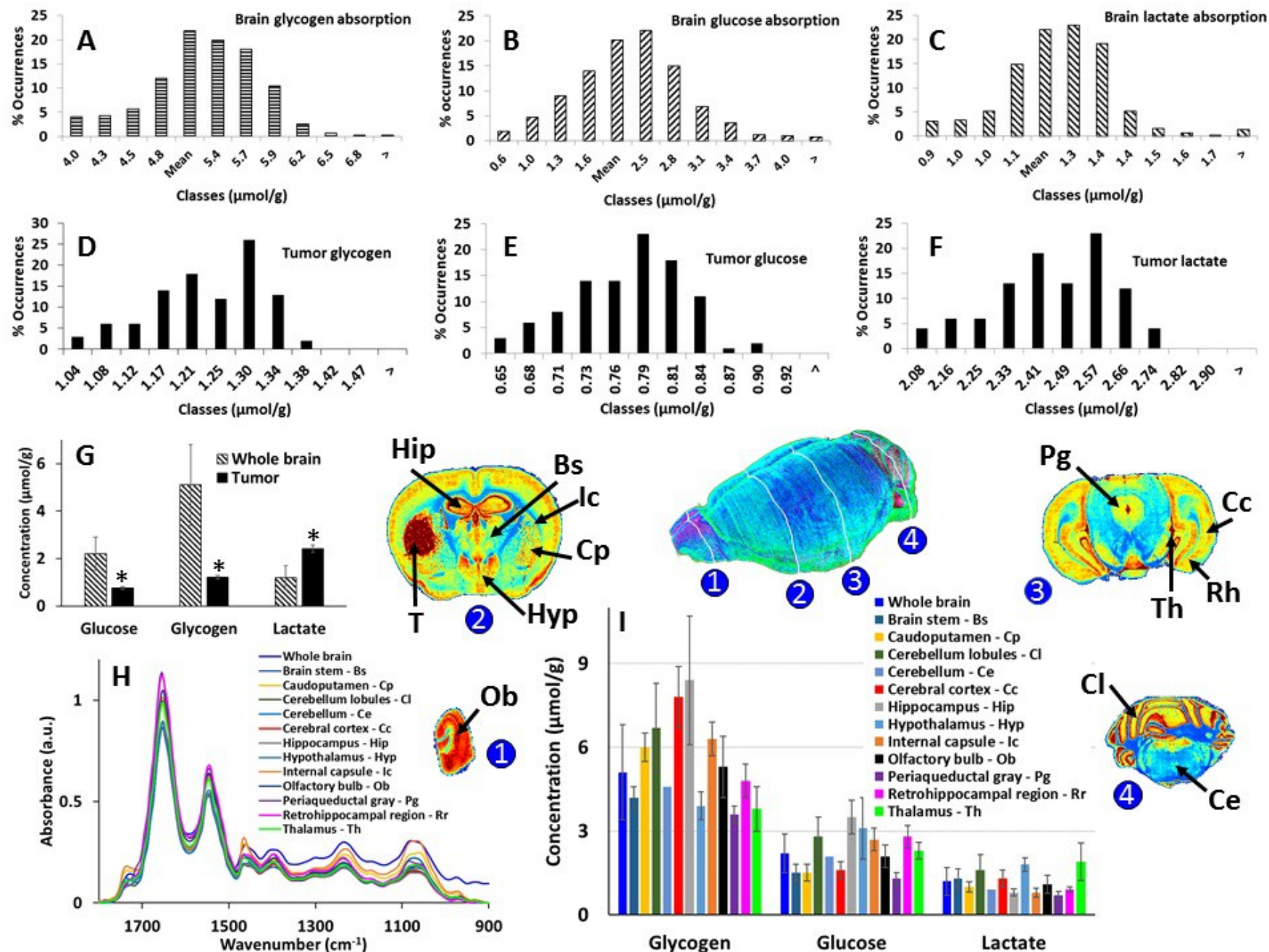

Supplement: Supplementary file 5 [file SC-009-C7SC03306K-s005.pdf]
