# Supplementary material for: 3D chemical imaging of the brain using quantitative IR spectro-microscopy
Source: Chem Sci. 2017 Oct 17;9(1):189–98. doi: 10.1039/c7sc03306k (PMC5869290; doi:10.1039/c7sc03306k)
Supplement: Supplementary file 6 [file SC-009-C7SC03306K-s006.pdf]

Electronic Supplementary Material (ESI) for Chemical Science.
This journal is © The Royal Society of Chemistry 2017

Supplementary Information

3D chemical imaging of the brain by quantitative IR spectro-microscopy

Abiodun Ogunleke[1], Benoit Recur[1], Hugo Balacey[1], Hsiang-Hsin Chen[2], Maylis Delugin[1], Yeukuang Hwu[2], Sophie Javerzat[1], Cyril Petibois[1,2]

1: University of Bordeaux, Inserm U1029 LAMC, Allée Geoffroy Saint-Hilaire Bat. B2, F33600 Pessac, France
2: Academia Sinica, Institute of Physics, 128 Sec. 2, Academia Rd., Nankang, Taipei 11529, Taiwan R.O.C.

Supplementary material 1: X-ray tomogram of the mouse brain in skull. The movie is obtained from 360 projections (from -90 to 90 degrees with respect to the frontal view of the head).

Supplementary material 2: From *in situ* X-ray high-resolution tomogram to *in vivo* X-ray radiographs. To reduce the X-ray dose applied to the animal before 3D histology by IR spectro-microscopy, a high-resolution model of the mouse brain (here a rag-γ immunodeficient mouse) is meshed from X-ray tomogram. It allows refining the brain and skull anatomical metrics at few microns spatial resolution. Further, for a histological animal experiment, 3 *in vivo* radiographs planes - coronal, axial, sagittal - and associated 2D metrics allow reconstructing a 3D model of mouse brain. The brain can be further used for histological analyses and 3D reconstruction without alteration of its chemical contents. The 3D model of the mouse brain will be further used for virtual sectioning and correction of histological images.

Supplementary material 3: Three-dimensional representations of the brain inside the skull after segmentation of the 370 projections obtained for the X-ray tomogram. The videos shows the X-ray tomogram of the actual mouse brain volume, with and without skull.

Supplementary material 4: The videos shows the resizing and 3D patch of the 100 anatomical images from the "Allen Mouse Brain Atlas", with skull (Image credit: Allen Institute). This resizing of 2D images will be further used for co-registration and correction of IR and histological images to achieve the reconstruction of 3D images.

Supplementary material 5: IR images obtained from a mouse brain. (A): The 2D-IR image of the section shown is approximatively located at Bregma -4.04 mm / internaural -0.24 mm. The mouse brain has been sectioned at 20-µm thickness (370 sections). The IR image is shown as a full spectral absorbance (in a.u.^{2}) and IR spectra in (B) corresponding to the 1-4 positions are shown in the central panel. They show important absorbance intensity variations throughout the spectral interval (1800-900 cm^{-1}, also called the fingerprint region). These absorbance intensity variations allow recognizing the major anatomical regions of the mouse brain, which can be used for proper alignment using anatomical atlas of the brain (such as the Allen Developing Mouse Brain Atlas). Distinctively, we can notice the areas corresponding to the white and gray (cortical and inner) matter of the brain.

Supplementary material 6: 3D-IHC image of the mouse brain and tumor.

Supplementary material 7: The 3D-IR image obtained using the [∫(1760-1710 cm^{-1})/∫(1700-1592 cm^{-1}) *100] absorption ratio to highlight the tumor mass. Noticeably, we can see the white matter distribution (purple-blue part of the brain image) crossed by the tumor (developed from inner to cortical gray matter and crossing white matter).

Supplementary material 8: Video of the tumor volume extracted from the 3D-IR and 3D-IHC images. In the 3D-IHC image, the green channel of the 3D histological image is segmented to extract the tumor volume. The videos of the IR and IHC brain and tumor volume renderings are shown side-by-side for visual comparison.

Supplementary Materials 9-11: 3D quantitative analysis of brain metabolism. The 3D histological images of the brain show the distribution (with classes according to SD around the mean value) of glucose (8a), glycogen (8b), and lactate (8c) concentrations. 3D images are scaled with equivalent absorption ($a.u.^2 .10^{-4}$) and concentration (µmol/g) values. The tumor volume has been meshed to highlight the quantitative differences with respect to healthy tissues (but maintaining the color gradients corresponding to its concentration values on the image scale).

Supplementary Material 12: Quantitative brain metabolism per region. A-F: The scaling of molecular concentrations has been performed using the left hemisphere of the brain as a healthy reference tissue. The distribution of IR absorptions allows defining the scale of concentrations within tissues at a microscopic resolution. G: The analysis of tissue metabolic concentrations in different regions of the brain shows important heterogeneities, glucose scale ranging 1.3 to 3.5 µmol/g, glycogen 3.6 to 8.4 µmol/g, and lactate 0.7 to 1.9 µmol/g. Conversely, the calculation of metabolic concentrations within the tumor mass showed limited heterogeneity, with 0.8 ± 0.1 µmol/g for glucose, 1.2 ± 0.1 µmol/g for glycogen, and 2.4 ± 0.2 µmol/g for lactate (significantly different from global brain values in healthy brain; $P<0.05$).

Supplementary materials 13-14: Videos of the 3D patch of 370 2D-IR images of the mouse brain sections without (13) and after (14) shape corrections. In 13, the images are aligned according to the central axis defined by the longitudinal cerebral fissure of the cortex and its center.
